# Characterizing CD38 Expression and Enzymatic Activity in the Brain of Spontaneously Hypertensive Stroke-Prone Rats

**DOI:** 10.3389/fphar.2022.881708

**Published:** 2022-05-31

**Authors:** Yousef Hannawi, Mohamed G. Ewees, Jordan T. Moore, Jay L. Zweier

**Affiliations:** ^1^ Division of Cerebrovascular Diseases and Neurocritical Care, Department of Neurology, The Ohio State University, Columbus, OH, United States; ^2^ Division of Cardiovascular Medicine, Department of Internal Medicine, Davis Heart and Lung Research Institute, The Ohio State University, Columbus, OH, United States; ^3^ Department of Pharmacology and Toxicology, College of Pharmacy, Al-Azhar University, Cairo, Egypt; ^4^ Department of Biomedical Engineering, The Ohio State University, Columbus, OH, United States

**Keywords:** cerebral small vessel disease, hypertension, oxidative stress, spontaneously hypertensive stroke prone rat, nitric oxide, nitric oxide synthase

## Abstract

**Background:** CD38 is a transmembrane glycoprotein that catabolizes nicotinamide adenine dinucleotide (NAD^+^) and is the main source for the age-dependent decrease in NAD^+^ levels. Increased CD38 enzymatic activity has been implicated in several neurological diseases. However, its role in the pathogenesis of cerebral small vessel disease (CSVD) remains unknown. We aimed to characterize CD38 expression and enzymatic activity in the brain of spontaneously hypertensive stroke-prone rats (SHRSP), a genetic model for hypertension and human CSVD, in comparison to age-matched normotensive Wistar Kyoto rats (WKY).

**Materials and Methods:** Age-matched male 7- and 24-week-old WKY and SHRSP were studied. CD38 enzymatic activity was determined in the brain homogenate. Immunohistochemistry and Western Blotting (WB) were used to characterize CD38 expression and localize it in the different cell types within the brain. In addition, expression of nitric oxide synthase (NOS) isoforms and the levels of nitric oxide (NO), superoxide, nicotinamide dinucleotide (phosphate) NAD(P)H were measured the brain of in WKY and SHRSP.

**Results:** CD38 expression and enzymatic activity were increased in SHRSP brains compared to age matched WKY starting at 7 weeks of age. CD38 expression was localized to the endothelial cells, astrocytes, and microglia. We also identified increased CD38 expression using WB with age in SHRSP and WKY. CD38 enzymatic activity was also increased in 24-week SHRSP compared to 7-week SHRSP. In association, we identified evidence of oxidative stress, reduced NO level, reduced NAD(P)H level and endothelial NOS expression in SHRSP compared to age matched WKY. NAD(P)H also decreased with age in WKY and SHRSP. Additionally, activation of astrocytes and microglia were present in SHRSP compared to WKY.

**Conclusions:** CD38 is overexpressed, and its enzymatic activity is increased in SHRSP, a genetic model for marked hypertension and human CSVD. Our results suggest a potential role for CD38 enzymatic activation in the pathogenesis of CSVD and points to the need for future mechanistic and pharmacological studies.

## Introduction

Cerebral Small Vessel Disease (CSVD) is a major public health burden resulting in intracerebral hemorrhage (ICH), vascular cognitive impairment (VCI) and acute ischemic stroke of lacunar type (Pantoni, 2010; Wardlaw et al., 2019). Age and hypertension are considered the main risk factors for CSVD (Pantoni, 2010; Hannawi et al., 2018; Wardlaw et al., 2019; Hannawi et al., 2021a). The brain of elderly patients with CSVD show signs of demyelination, lacunar formation, microbleed, and enlarged perivascular spaces (Wardlaw et al., 2013). To study the underlying mechanisms of disease and develop targeted therapeutics, hypertensive animal models have been utilized (Bailey et al., 2011a; Mustapha et al., 2019). In particular, spontaneously hypertensive stroke-prone rats (SHRSP) have been the most frequently used animal model for CSVD (Bailey et al., 2011a; Schreiber et al., 2013). Previous research from other groups and ours have shown progressive development of neuroimaging and histopathological CSVD features in SHRSP as they age (Schreiber et al., 2012; Schreiber et al., 2013; Hannawi et al., 2021b). From pathophysiological and mechanistic perspectives, the brains of SHRSP and humans with CSVD show evidence for oxidative stress, endothelial dysfunction, impairment of endothelial nitric oxide synthase (eNOS) with decreased nitric oxide (NO) production, and blood brain barrier (BBB) breakdown [(Schreiber et al., 2013; Mustapha et al., 2019), (Qadri et al., 2003; Rajani et al., 2018; Zhang et al., 2019)]. However, the main mechanisms leading to these findings including endothelial dysfunction and oxidative stress in both human and SHRSP are still unknown.

CD38 is a transmembrane glycoprotein that functions as an ectoenzyme in catabolizing nicotinamide dinucleotide (NAD^+^) (Hogan et al., 2019). Recent work has suggested that CD38 enzymatic activity increases with aging, and it is the main determinant of the age-dependent decrease in NAD^+^ levels (Camacho-Pereira et al., 2016). These results have sparked interest in linking CD38 enzymatic activity with various age-dependent neurological diseases such as neurodegeneration and dementia (Guerreiro et al., 2020). In addition to the age-dependent increase in CD38 enzymatic activity, our research group has identified other mechanisms for CD38 activation that are seen in the setting of coronary ischemia/reperfusion which results in worsening of the ischemic cardiac injury (Boslett et al., 2018a; Boslett et al., 2018b). In these models, oxidative stress induced an increase in CD38 enzymatic activity leading to NAD(P)H depletion and endothelial dysfunction (Reyes et al., 2015; Boslett et al., 2018a). Administration of a potent CD38 inhibitor resulted in reversal of endothelial dysfunction and improvement of post ischemic cardiac injury (Boslett et al., 2017; Boslett et al., 2019).

Hypertension is another condition that is known to be associated with oxidative stress, which is in turn implicated in the pathophysiology of hypertensin-induced end organ dysfunction including hypertension-induced brain dysfunction and CSVD (Poulet et al., 2006; Loperena and Harrison, 2017). However, to date, it is not known whether CD38 enzymatic activity is increased in the setting of hypertension induced CSVD and oxidative stress (Reyes et al., 2015). Therefore, in this work, we characterize CD38 expression and enzymatic activity in the brain of SHRSP, a genetic model of severe hypertension and CSVD, compared to age-matched normotensive Wistar-Kyoto (WKY) rats. Our primary hypothesis is that CD38 expression and enzymatic activity are increased in the brain of SHRSP compared to age matched WKY and this occurs prior to the detection of significant CSVD lesions. We observe that the expression of CD38 is present on endothelial cells, astrocytes, and microglia with increased CD38 levels and enzymatic activity in the brain of SHRSP compared to age matched WKY. In association, the brains of SHRSP show decreased NAD(P)H and NO levels with accompanying evidence of oxidative stress. These findings are indicative of a potential mechanistic role of CD38 in hypertension induced CSVD and suggest that CD38 may be an important therapeutic target for future interventional studies.

## Materials and Methods

### Experimental Study Design

All the study procedures were approved by the Institutional Animal Care and Use Committee (IACUC) at The Ohio State University, and they were conducted in compliance with the Public Health Service Policy on Humane Care and Use of Laboratory Animals. The experiments are reported in compliance with ARRIVE guidelines. Equal number of male SHRSP and male age matched WKY were obtained from Charles River Laboratories (Wilmington, MA) at 5 weeks of age. Male rats are used in this experiment as previous studies showed that they develop higher blood pressure with consistent CSVD lesion formation compared to female rats (Bailey et al., 2011a). All animals were kept under the same physiological conditions, and they were housed 2 rats per cage. Rats had unlimited access (ad libitum) to regular lab chow (Terklad LM-485 Mouse/Rat Serializable Diet; 19.1% protein, 0.3% sodium and 0.8% potassium) that was obtained from Envigo, Inc. with free access to tap water without additional dietary salt. The rats were randomly divided into 2 experimental groups, each containing equal numbers of rats at the beginning of the study (10 WKY and 10 SHRSP). One group was euthanized at 7 weeks of age and the second group was followed until they reached 24 weeks of age. The rat groups and timepoints in this current study were selected carefully from our previous longitudinal study of the natural history of CSVD in SHRSP showing near absence of CSVD lesions, except for mildly enlarged perivascular spaces, at 7 weeks of age and established CSVD lesions at 24 weeks of age (Hannawi et al., 2021b). The main hypothesis of our current study was that an increase in CD38 expression and enzymatic activity would occur prior to CSVD progression, for which 7-week old SHRSP and WKY were used, and they would persist or increase in later life, for which 24-week old SHRSP and WKY were selected. Euthanasia was completed for all rats in the study upon reaching the pre-specified study time points. All animals were monitored weekly for signs of stroke or intracerebral hemorrhage including unilateral weakness, decreased movement, seizures, or significant weight loss. Weight was monitored weekly using a standard weighing scale. Animals that had greater than 20% weight loss were euthanized according to IACUC regulations.

### Blood Pressure Measurements

The systolic blood pressure (SBP) was measured in the non-sedated rats using cuff-tail plethysmography device (Visitech Systems, Inc.) starting at 6 weeks of age and it was repeated weekly in most rats until they reached the study timepoints. A series of 10 measurements were performed at each session following a 10-min acclimation period in the device. The median of SBP measurements were calculated for each session.

### Euthanasia and Tissue Processing

Upon reaching the study time point, euthanasia was performed using CO_2_ inhalation followed by decapitation. Subsequently, the brain was extracted and carefully washed in PBS solution for 2 min. A standard brain matrix was used to section the brain into three parts. The anterior part of the brain was snap frozen in liquid nitrogen for CD38 enzymatic activity assay. The middle part of the brain was embedded in optimal cutting temperature (OCT) compound and snap frozen using liquid nitrogen for subsequent immunohistochemistry (IHC) processing. The final part underwent standard fixation in formaldehyde for 48 h and subsequent paraffin processing. Sections for Hematoxylin and Eosin (H&E) staining were performed from the processed paraffin block at the level of the posterior corpus callosum and hippocampus to assess for the development of CSVD lesions. Slides were scanned using Axio Scanner system (Carl Zeiss, Inc. Oberkochen, Germany). The slides were assessed for CSVD lesions including enlarged perivascular spaces, microbleeds, demyelination, hemosiderin deposition and lacunes as described in previous studies (Deramecourt et al., 2012; Hannawi et al., 2021b).

### Immunohistochemistry Staining

Brain sections for IHC staining were first washed with PBS to dissolve the residual OCT. Then, sections were fixed with 4% paraformaldehyde, washed briefly with PBS, and blocked with 5% Blocker^TM^ BSA in PBS containing 0.3 M glycine. After 1 h of blocking at room temperature, the primary antibodies were added for overnight incubation at 4°C. Supplementary Table S1 contains the list of the antibodies that were used in this study along with their concentration and sources. Following overnight incubation, sections were washed 3 times for 5 min with PBS and secondary antibodies were added for 30 min incubation period at room temperature in the dark. Anti-fade mounting media (Southern Biotechnology Associates, Birmingham, AL, United States) containing the nuclear stain 4′,6-Diamidino-2-Phenylindole (DAPI; 1 μM) was applied. Brain sections were digitally imaged and different fields were captured at 40× using a confocal microscope (FV3000 spectral confocal microscope, Tokyo, Japan). Fluorescence intensity was analyzed using the software associated with the microscope for at least 5 sections per animal.

To characterize the expression of CD38 on the several types of cells present in the brain of WKY or SHRSP, we performed co-staining with primary antibodies that bind to specific components of these cells with and without CD38 antibodies. Several slides were separated following the addition of CD38 primary antibody and co-staining with eNOS, GFAP, Iba1, CD3, CD68, MPO, and NeuN antibodies were performed to delineate CD38 expression on endothelial cells, glial cells, microglia, T-cells, macrophages, neutrophils, and neurons, respectively. Following addition of these primary antibodies the rest of the IHC staining steps were similarly followed. Images were taken on an Olympus FV 1000 spectral confocal microscope with a × 60 objective. For nuclei staining, DAPI was used at a concentration of ∼1 μM.

### Western Blotting for CD38

Frozen brain tissue was homogenized in ice-cold RIPA lysis buffer and centrifuged. Supernatant was collected and protein concentration was determined. Cellular proteins from brain tissue homogenates were separated into a gradient 4–20% SDS- polyacrylamide gel (Bio-Rad, Hercules, CA, United States), and electro-blotted on PVDF membranes. CD38 was detected using a mouse monoclonal anti-CD38 antibody and GAPDH using rabbit monoclonal anti-GAPDH (Supplementary Table S1), in 1% non-fat dry milk in TBST overnight at 4°C. Membranes were washed with TBST and incubated with corresponding HRP linked secondary antibody (Supplementary Table S1) in 1% non-fat dry milk in TBST for 1 h at room temperature, followed by rocking for 30 min with three changes of TBST. Amersham ECL Western Blotting Detection Reagent (GE Healthcare, Chicago, IL, United States) was used to develop the membranes. Protein expression was quantitated from the blots, using ImageJ software. Bands were normalized to GAPDH band intensity.

### CD38 Enzymatic Activity Assay

CD38 functions as an NAD(P)^+^ase through its hydrolysis of NAD(P)^+^ to 2′-P-ADPR. To measure this enzyme activity specifically, a substrate analog of NAD^+^, nicotinamide 1,N^6^-ethenoadenine dinucleotide (ε-NAD) was used. WKY and SHRSP brains were homogenized in buffer containing 150 mM NaCl, 50 mM Tris, 1 mM EDTA, 1% Triton X-100, and freshly added protease inhibitors. Homogenate totaling 100 μg of protein was added to a 200 μl reaction mixture containing 200 μM ε-NAD. Reactions were monitored for the conversion of ε-NAD to strongly fluorescent product etheno-ADP-ribose (ε-ADPR). Fluorescence was measured at an excitation wavelength of 300 nm and an emission wavelength of 410 nm on a Molecular Devices SpectraMax plate reader.

### *In Situ* Superoxide Detection

Staining of 5 µM sections of the frozen brains with dihydroethidium (DHE) and DAPI was performed in the dark to determine superoxide generation in the brain sections. Incubation of additional sections with 100 μM of the SODm, MnTBAP (Santa Cruz Biotechnology Inc., Santa Cruz, CA, United States) for 10 min prior to DHE was applied in matched control experiments to confirm the specificity of the fluorescence and prove that signals are derived from superoxide. The slides were rinsed extensively with PBS, mounted in antifade mounting media Fluoromount-G (Southern Biotechnology Associates, Birmingham, AL, United States), cover slipped, then different fields were captured at ×40 using a confocal microscope (Olympus FV3000 spectral confocal microscope, Tokyo, Japan) with excitation wavelength at 495 nm and emission at 515 nm, and the fluorescence intensity was analyzed using the microscope provided software.

### *In Situ* Nitric Oxide Detection

The NO-specific fluorescent dye 4,5-diaminofluorescein diacetate (DAF-2 DA) was used as a measure of NO. Five µm sections were made from the frozen OCT blocks and stained with 5 μM DAF-2 DA and DAPI in PBS for 30 min in dark. In matched control experiments, the sections were incubated with 100 μM of the NO scavenger, PTIO (Sigma, Burlington, MA, United States) for 10 min prior to DAF-2 DA to confirm the specificity of the fluorescence and prove that signals are derived from NO. The slides were rinsed extensively with PBS, mounted in antifade mounting media Fluoromount-G (Southern Biotechnology Associates, Birmingham, AL, United States), cover slipped, then different fields were captured at ×40 using a confocal microscope (Olympus FV3000 spectral confocal microscope, Tokyo, Japan) with excitation wavelength at 500–530 nm and emission at 590–620 nm, and the fluorescence intensity was analyzed using the microscope provided software.

### *In Situ* Detection of NAD(P)H

JZL1707 NAD(P)H sensor (AAT Bioquest, Sunnyvale, CA, United States) was used to incubate 5 µM section slides at 37°C for 30 min according to manufacturer instructions. The slides were rinsed with PBS, mounted in antifade mounting media Fluoromount-G (Southern Biotechnology Associates, Birmingham, AL, United States), cover slipped, then different fields were captured at 40x using a confocal microscope (Olympus FV3000 spectral confocal microscope, Tokyo, Japan) with excitation wavelength at 540 nm and emission at 590 nm, and the fluorescence intensity was analyzed using the microscope provided software.

### Statistical Analysis

Statistical analysis was completed in Microsoft Excel and GraphPad Prism. Means and standard deviations were used to represent the continuous variables. We used standard T-Test to compare SBP between WKY and SHRSP. To test our primary hypothesis that CD38 expression and enzymatic activity are increased in the brain of SHRSP in comparison to WKY prior to CSVD, we used also T-test for this purpose by comparing CD38 expression and enzymatic activity between WKY and SHRSP at 7 weeks of age. As CD38 expression and enzymatic activity have also been shown in the literature to increase with aging, we secondarily explored whether there is an interaction between the rat type (WKY vs SHRSP) and age in our analysis using two-way ANOVA test. This analysis allowed us to determine whether an interaction was present and determine the main effect of the rat type and age on all of the measured metrics. We subsequently used Fisher’s Least Significant Difference (LSD) test post ANOVA to measure the difference among the individual groups. We did not adjust for multiple comparisons in this analysis as it was exploratory in nature aiming to quantify the differences among the group following testing our specific primary hypothesis. *p* value of < 0.05 was considered statistically significant for all comparisons. Results of Fisher’s LSD were reported in the figures.

## Results

40 male rats (20 WKY and 20 SHRSP) were used in this study. 20 rats (10 WKY and 10 SHRSP) were euthanized at 7 weeks of age while the remaining 20 were followed until they reached 24 weeks of age. None of the animals had significant weight loss or signs of stroke during the experiment.

### Systolic Blood Pressure

**FIGURE 1:**
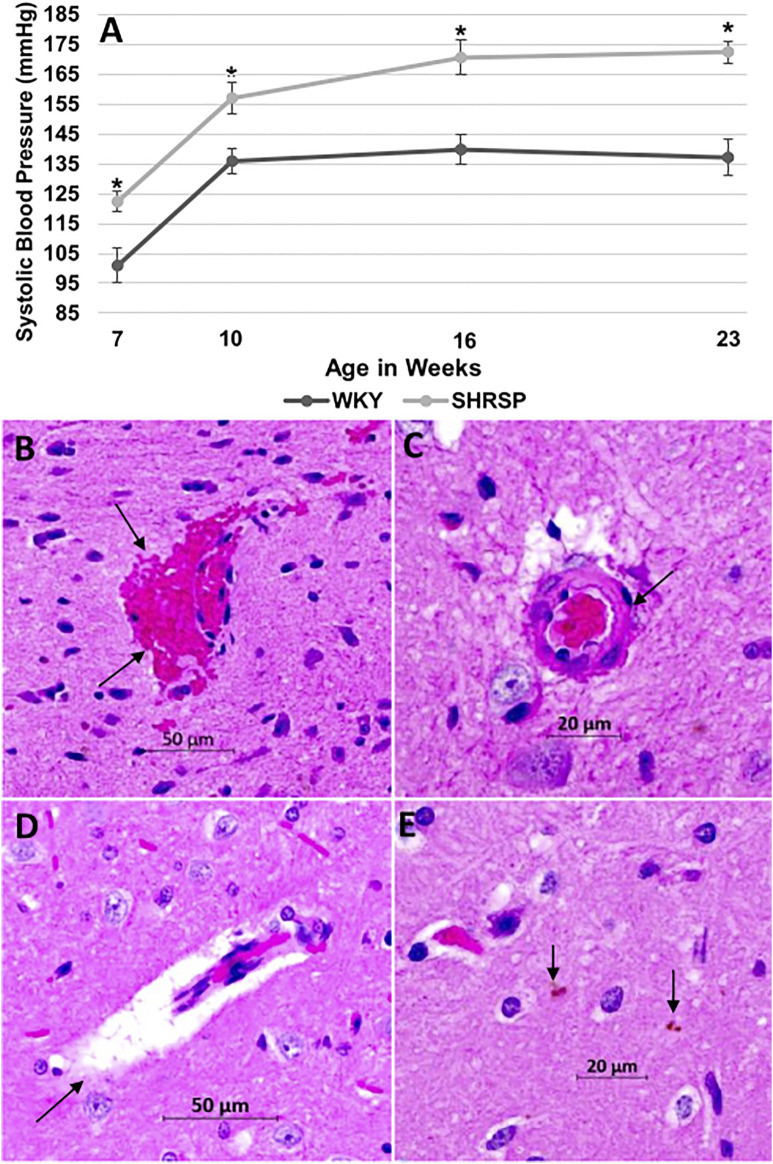
SHRSP develop hypertension and CSVD lesions as they age. The temporal changes of SBP in WKY and SHRSP at 7, 10, 16, and 23 weeks of age are presented in **(A)**. SBP was significantly higher in SHRSP compared to WKY throughout the study. At 7 weeks of age, SHRSP were not yet hypertensive. SHRSP subsequently developed hypertension and sustained it afterwards. Data represent means and standard errors, (*) P < 0.01 compared to WKY. The histological findings of CSVD are shown on Hematoxylin and Eosin (H&E) staining at ×20 in the brains of SHRSP **(B–E)**. Brain sections were obtained at the level of the posterior corpus callosum and hippocampus. At 24 weeks of age, SHRSP brains showed evidence of capillary congestion with red blood cells extravasation and microbleed formation (arrows in B), vessel wall thickening and lipohyalinosis (arrow in C), dilatation of the perivascular spaces (arrow in D), and small hemosiderin depositions (arrows in E). CSVD: cerebral small vessel disease; SBP: systolic blood pressure; mmHg: millimeter mercury; SHRSP: spontaneously hypertensive stroke-prone rats; WKY: Wistar Kyoto rats.

The SBP was significantly higher in SHRSP compared to WKY throughout the experiment (Figure 1A). At the beginning of the experiment, SHRSP were still not hypertensive but had higher SBP than WKY (SBP at 7 weeks: WKY 101 ± 19 mmHg vs. SHRSP 122 ± 11 mmHg, *p* = 0.007). Subsequently, SHRSP developed chronic hypertension with sustained elevation in SBP compared to WKY (SBP at 10 weeks: WKY 136 ± 13 mmHg vs. SHRSP 157 ± 17 mmHg, *p* = 0.0058). This chronic hypertension was maintained throughout the remainder of the experiment (SBP at 23 weeks: WKY 137 ± 16 vs. SHRSP 172 ± 10, *p* = 0.0006).

### Histopathological Changes of Cerebral Small Vessel Disease

Hematoxylin and Eosin (H&E) staining of WKY brains did not reveal histological findings of CSVD. In SHRSP, however, the most consistent finding of CSVD at 7 weeks of age was mild dilatation of the perivascular spaces (PVS). At 24 weeks of age, the brains of SHRSP showed consistent histological changes with CSVD that were prevalent in all SHRSP including capillary congestion, RBCs extravasation with microbleed formation (Figure 1B), wall thickening of the small and medium vessels with lipohyalinosis (Figure 1C), enlargement of PVS (Figure 1D), and hemosiderin depositions (Figure 1E).

### Measurement of CD38 Expression and Enzymatic Activity

**FIGURE 2:**
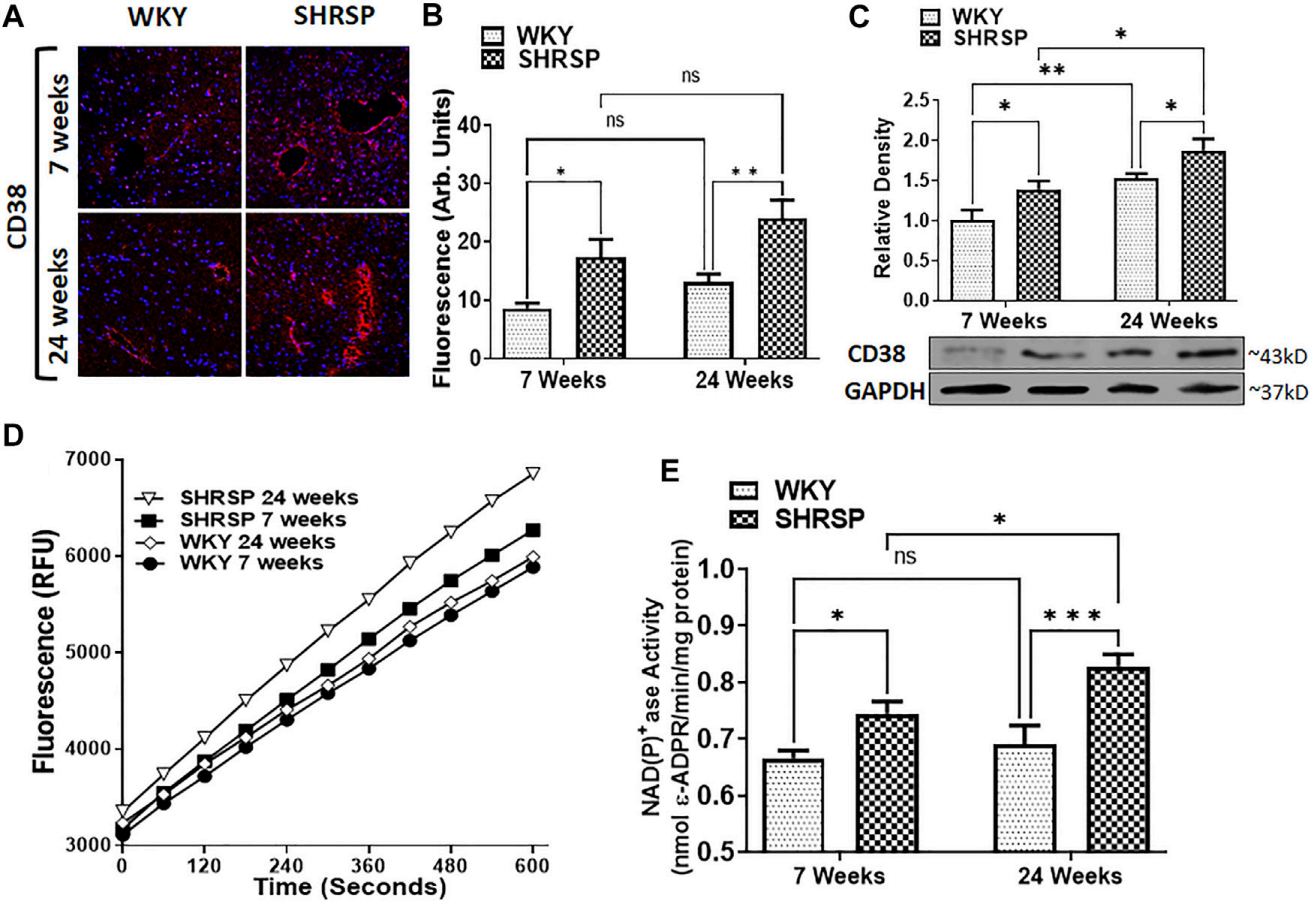
CD38 expression using Immunohistochemistry (IHC) staining for CD38 antibody and DAPI **(A,B)**, Western Blotting **(C)**, and CD38 enzymatic activity **(D,E)** in the brains of WKY and SHRSP. Group comparisons are completed in the figure using Fisher’s LSD test post ANOVA. Results indicate significantly increased CD38 expression and enzymatic activity in SHRSP compared to WKY at 7 and 24 weeks of age. Differences were also detected in some comparisons according to age as indicated in the figure. DAPI: 4^’^,6-diamidino-2-phenylindole; SHRSP: spontaneously hypertensive stroke-prone rats; WKY: wistar-kyoto rat; ns: not statistically significant; (*) *p* < 0.05, (**) *p* < 0.01, (***) *p* < 0.001. Data represent the means and standard errors.

CD38 expression was detected by IHC fluorescence and WB (Figures 2A, 2B, and 2C). The specificity of anti-CD38 antibody used in our experiment is shown in Supplementary Figure S1. In testing our primary hypothesis, CD38 expression and enzymatic activity were significantly higher in the brain of SHRSP compared to WKY at 7 weeks of age (IHC: 103% higher, *p* = 0.011, WB: 39% higher, *p* = 0.049 and CD38 enzymatic activity was 12% higher, *p* = 0.012). ANOVA testing was negative for interaction between the age and type of rats in all IHC, WB and enzymatic activity results (*p* = 0.65, 0.88, and 0.22, respectively). However, it showed a significant main effect for the rat type (higher CD38 expression in SHRSP by IHC and WB, *p* = 0.0002, 0.007, respectively and higher CD38 enzymatic activity in SHRSP, *p* = 0.0002) and age (higher CD38 expression and enzymatic activity in 24 weeks rats including IHC: *p* = 0.023, WB *p* = 0.0009, and CD38 enzymatic activity: *p* = 0.037). Individual group comparisons using Fisher’s LSD are shown in Figure 2B for IHC, Figure 2C (WB), Figure 2D and Figure 2E (CD38 enzymatic activity). The full WB results are shown in Supplementary Figure S2. In summary, in addition to the elevation of CD38 expression and enzymatic activity in SHRSP at 7 weeks of age that was tested in our primary hypothesis, the following statistically significant differences were noted: IHC: CD38 expression was 83% higher (*p* = 0.0025) in 24-week SHRSP vs. 24-week WKY, WB: CD38 expression was 23% higher (*p* = 0.048) in 24-week SHRSP vs. 24-week WKY, 52% higher in 24-week WKY vs. 7-week WKY (*p* = 0.0073), and 49% higher in 24-week SHRSP vs. 7-week SHRSP (*p* = 0.011). CD38 enzymatic activity was 20% higher (*p* = 0.0006) in 24 weeks SHRSP vs. 24 weeks WKY and 11% higher (*p* = 0.023) in 24 weeks SHRSP vs. 7 weeks SHRSP. CD38 enzymatic activity was 4% higher in 24-week WKY vs. 7-week WKY without statistical significance (*p* = 0.5).

### Characterizing CD38 Expression in the Brains of Wistar Kyoto Rats and Spontaneously Hypertensive Stroke-Prone Rats

**FIGURE 3:**
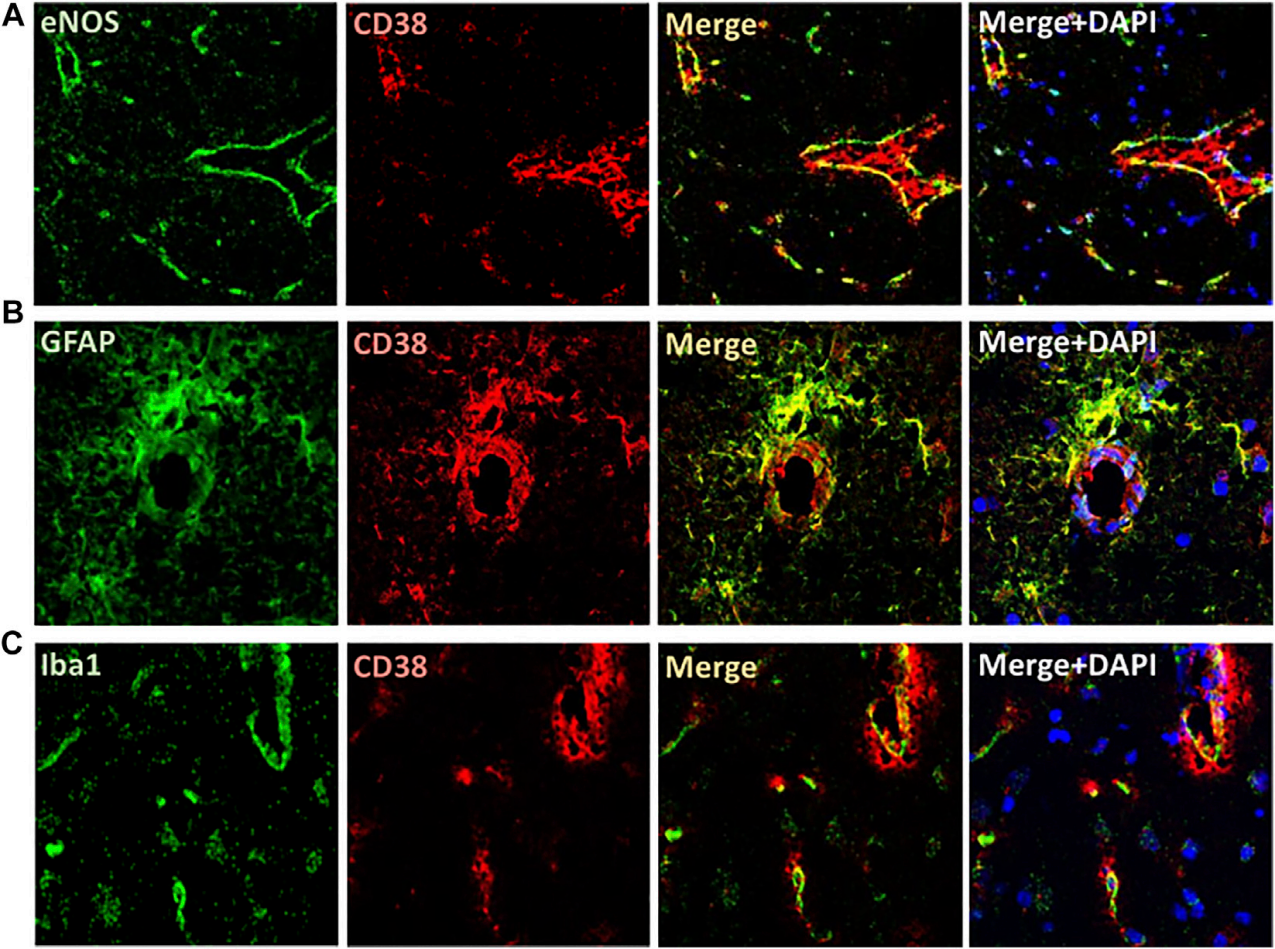
Localization of CD38 expression in the brain of SHRSP. Immunofluorescence is shown in the brain of 24-week-old rats. CD38 expression was seen on the endothelial cells **(A)** and astrocytes **(B)** as manifested by colocalizing with eNOS and GFAP antibodies, respectively. A lesser degree of expression was seen on the microglia using co-localization with Iba-1 **(C)**. eNOS: endothelial nitric oxide synthase; GFAP: Glial fibrillary acidic protein; Iba1: ionized calcium binding adaptor molecule 1; SHRSP: spontaneously hypertensive stroke-prone rats.

**FIGURE 4:**
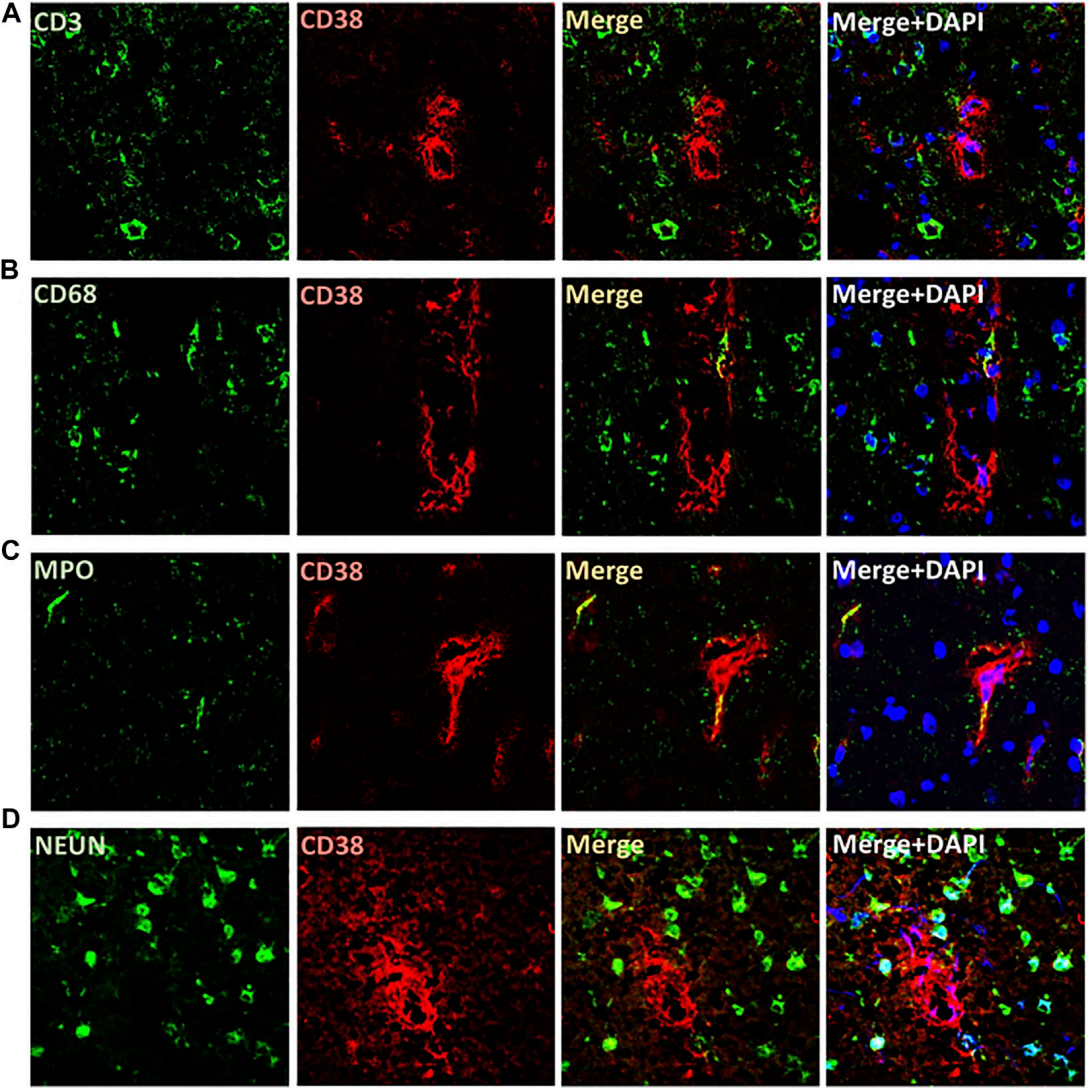
Localization of CD38 expression in the brains of SHRSP at 24 weeks of age. No expression was identified on T cells **(A)**, macrophages **(B)**, or neurons **(D)** by immunohistochemistry co-staining using CD38 and CD3 (T cells), CD68 (macrophages), and NEUN (neurons). Rare expression of CD38 was seen on neutrophils using co-staining with MPO **(C)**. MPO: myeloperoxidase; SHRSP: spontaneously hypertensive stroke-prone rats.

The expression of CD38 was seen on the astrocytes and endothelial cells in the brain. This was identified by co-staining of CD38 with GFAP and eNOS (Figures 3A,B). There was a lesser degree of CD38 expression seen on the microglia with co-staining of CD38 and Iba-1 (Figure 3C). CD38 expression was not seen in our model on the neurons, T-cells, or macrophages using co-staining with NeuN, CD3, CD68, respectively (Figures 4A,B,D). However, there was some expression of CD38 seen on neutrophils as determined by co-staining with MPO (Figure 4C).

### *In Situ* Measurement of NAD(P)H, Superoxide and Nitric Oxide

**FIGURE 5:**
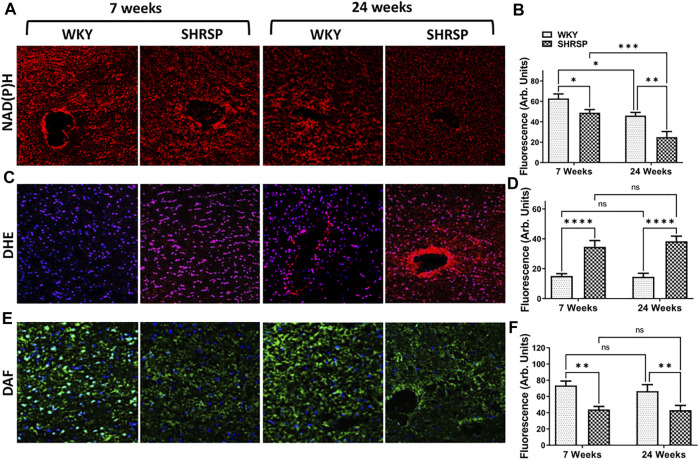
Brain of SHRSP shows evidence of oxidative stress and decreased NO and NAD(P)H. Staining for NAD(P)H **(A,B)**, superoxide (DHE + DAPI; **(C,D)** and nitric oxide (DAF + DAPI; **(E,F)** in WKY and SHRSP brains at 7 weeks and 24 weeks. Statistical comparisons were completed using Fisher’s LSD test post ANOVA. Significant differences consistent with lower NAD(P)H were seen in the brains of SHRSP compared to WKY and older rats compared to the young ones. Superoxide was significantly elevated in SHRSP compared to WKY at 7 and 24 weeks of age and NO was lower in SHRSP in comparison to WKY. No differences in superoxide and NO levels were detected according to age in WKY and SHRSP. DHE: Dihydroethidium; DAF: 4-amino-5-methylamino-2^’^,7^’^-Difluorofluorescein Diacetate; NAD(P)H: nicotinamide adenine dinucleotide phosphate; SHRSP: spontaneously hypertensive stroke-prone rats, WKY: wistar-kyoto rats; ns: not statistically significant; *: *p* < 0.05, **: *p* < 0.01, ***: *p* < 0.001, ****:*p* < 0.0001. Data represent the means and standard errors.

Given the increased enzymatic activity of CD38 in the brains of SHRSP compared to WKY, we subsequently examined NAD(P)H levels in the brains of SHRSP and WKY (Figure 5A). The results of the individual group comparisons are shown in Figure 5B. ANOVA testing was negative for interaction between the rat age and type (*p* = 0.41), but it showed significant main effect of lower NAD(P)H levels in the brain of SHRSP vs. WKY (*p* = 0.0005) and in 24-week-old vs. 7-week-old rats (*p* < 0.0001). Individual group comparisons showed that 24-week SHRSP had 57% lower NAD(P)H level than 24-week WKY (*p* = 0.002) and 59% lower than 7-week SHRSP (*p* = 0.0007). At 7 weeks of age, SHRSP had 22% lower NAD(P)H than same age WKY (*p* = 0.029). In addition, NAD(P)H was 27% lower in 24-week WKY than 7-week WKY (*p* = 0.01). DHE measurements of superoxide (Figure 5C) showed significantly higher levels in SHRSP vs. WKY (*p* < 0.0001) but no difference according to age (*p* = 0.62) using ANOVA. Similarly, there was no interaction between the age of rats and their type in this analysis (*p* = 0.49). Individual group comparisons are shown in Figure 5D where supeoxide levels were 132% higher in 7-week SHRSP vs. 7-week WKY (*p* < 0.0001) and 163% higher in 24-week SHRSP vs. 24-week WKY (*p* < 0.0001). There were no statistically significant differences in 7-week WKY vs. 24-week WKY and 7-week SHRSP vs. 24-week SHRSP. Similarly, there was no interaction between rat age and rat type in terms of NO level (*p* = 0.6) and the main effect of age on NO level was also not significant (*p* = 0.49). However, SHRSP had significantly lower NO levels in their brain compared to WKY (*p* < 0.0001). Individual group comparisons are shown in Figure 5F. 7-week SHRSP had 50% lower NO in comparison to 7-week WKY (*p* = 0.001) while 24 week SHRSP had 26% lower NO in comparison to 24-week WKY (*p* = 0.008).

### Detection of Nitric Oxide Synthase Isoforms, GFAP and Iba1 Expression

**FIGURE 6:**
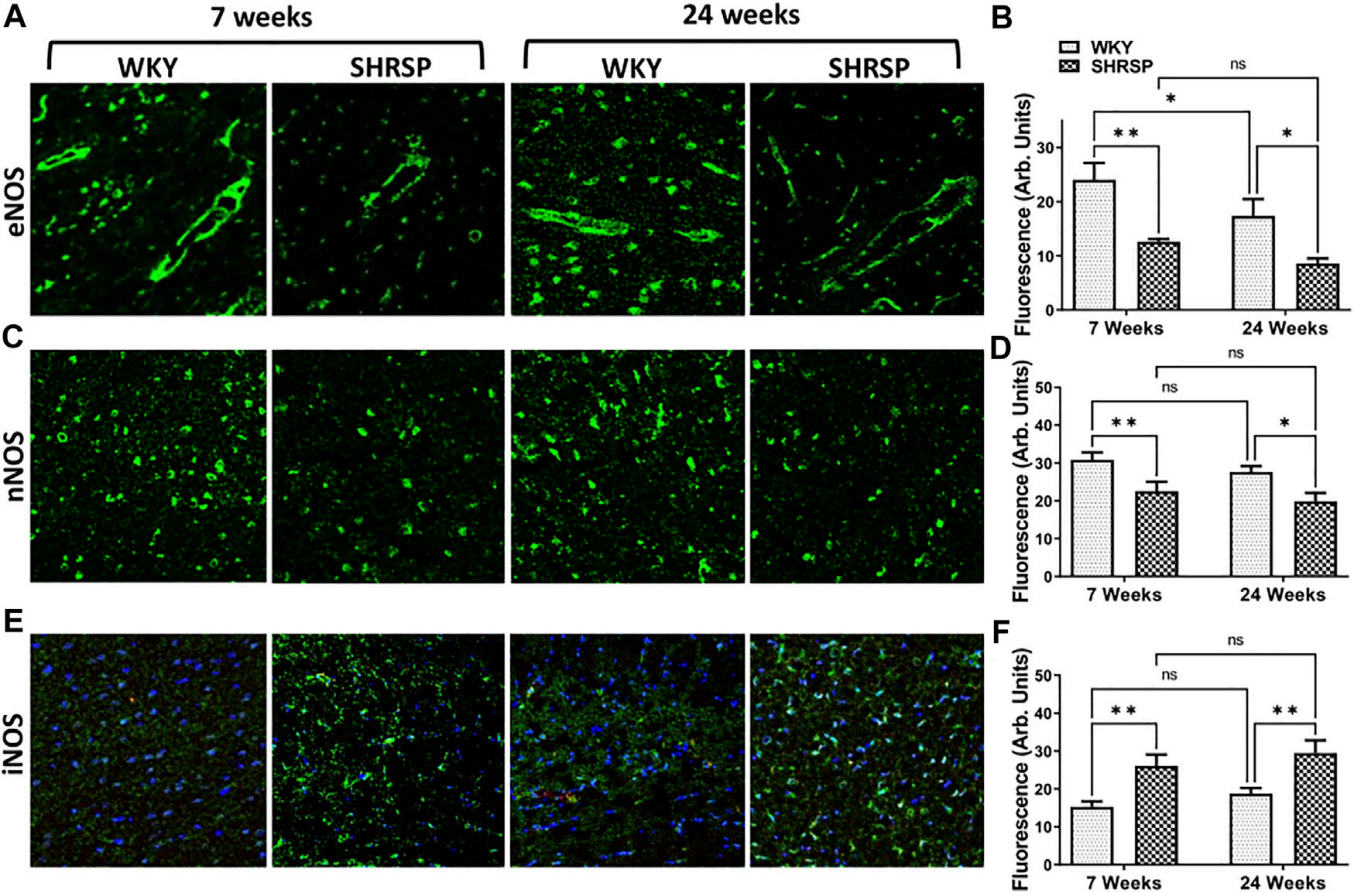
IHC staining for eNOS, nNOS, and iNOS in the brains of WKY and SHRSP. Significant differences consistent with lower eNOS and nNOS expression were detected in SHRSP in comparison to age matched WKY **(A–D)**. iNOS showed higher expression in SHRSP in comparison to WKY at the same time points **(E,F)**. eNOS was also significantly lower in 24-week WKY in comparison to 7-week WKY. There were no additional differences according to age in nNOS and iNOS expression. eNOS: endothelial nitric oxide synthase; iNOS: inducible nitric oxide synthase; nNOS: neuronal nitric oxide synthase; SHRSP: spontaneously hypertensive stroke-prone rats; WKY: Wistar Kyoto rats; ns: not statistically significant; *: *p* < 0.05; **: *p* < 0.01. Data represent the means and standard errors.

Since we identified a decrease in NO availability and an increase in superoxide in SHRSP compared to WKY, we examined the expression of NOS isoforms in the brains of SHRSP and WKY (Figure 6). We did not identify significant interaction between the rat type and age in any of these analyses (eNOS: *p* = 0.57, nNOS: *p* = 0.89 and iNOS: *p* = 0.99). In the case of eNOS (Figure 6A), there were significant differences in its expression according to the rat type (*p* = 0.0001) and age (*p* = 0.025). Individual group comparisons are presented in Figure 6B. In summary, eNOS expression was 48% lower in 7-week SHRSP vs. 7-week WKY (*p* = 0.0012) and 51% lower in 24-week SHRSP vs. 24-week WKY (*p* = 0.01). In addition, the expression of eNOS was 11% lower in 24-week WKY than 7-week WKY (*p* = 0.046). In the case of nNOS (Figure 6C), there were significant differences according to the rat type (*p* = 0.0007). However, there was no difference between WKY and SHRSP according to their age (*p* = 0.18). Individual group comparisons are shown in Figure 6D which showed 27% lower nNOS expression in 7-week SHRSP vs. 7-week WKY (*p* = 0.01) and 28% lower expression in 24-week SHRSP vs. 24-week WKY (*p* = 0.016). In contrast to this, the expression of iNOS (Figure 6E) was significantly higher in SHRSP vs. WKY (*p* = 0.0002). But there were no differences according to age (*p* = 0.2). Individual group comparisons are presented in Figure 6F which show 91% higher expression in 7-week SHRSP vs. 7-week WKY (*p* = 0.005) and 56% higher expression in 24-week SHRSP vs. 24-week WKY (*p* = 0.008).

**FIGURE 7:**
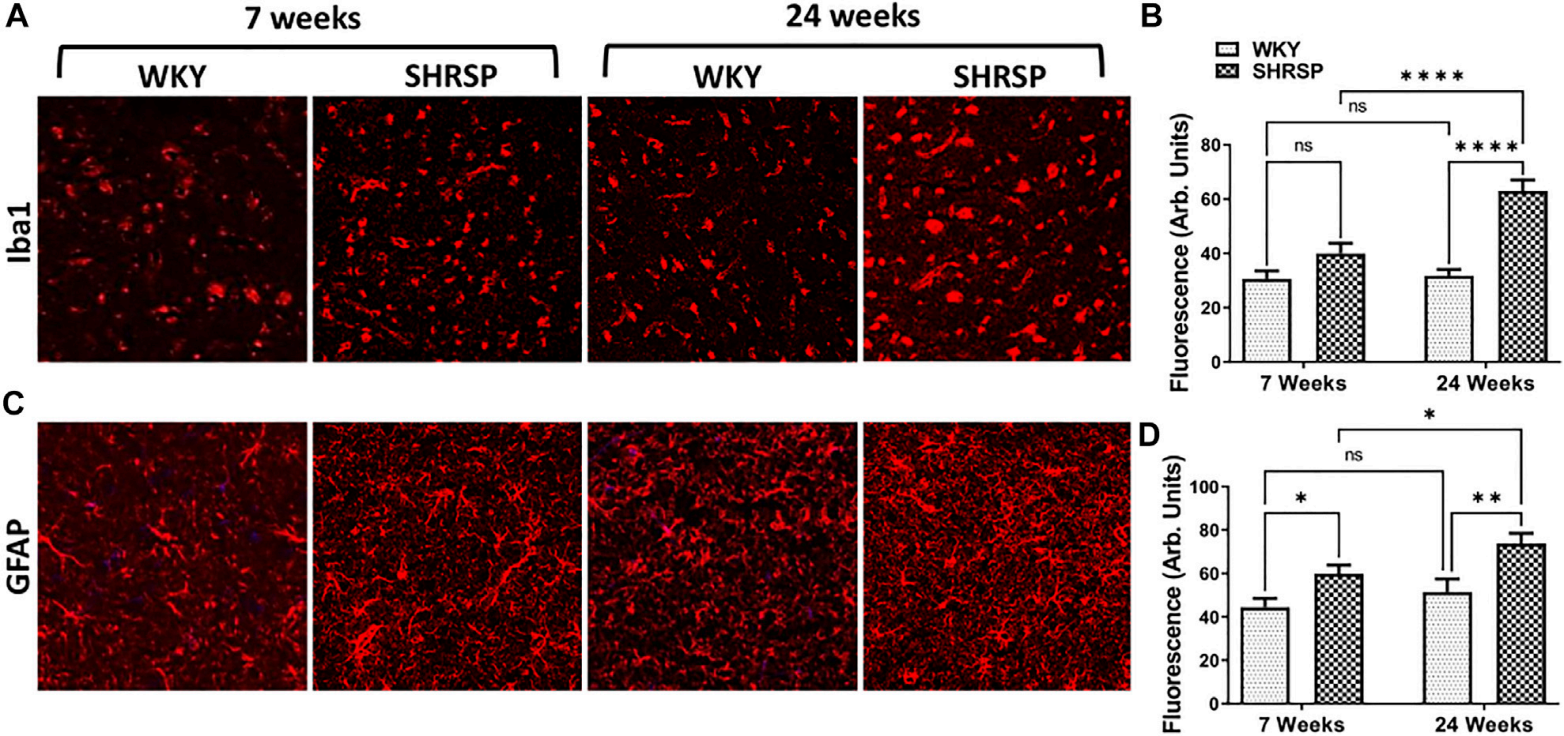
IHC staining for GFAP and Iba-1 in the brains of WKY and SHRSP.Iba1 **(A,B)** expression shows significant interaction for the rat age and type. 24-week SHRSP had significantly higher expression of Iba1 in comparison to 24-week WKY and 7-week SHRSP. GFAP expression **(C, D)** was significantly higher in 7-week SHRSP in comparison to 7-week WKY and 24-week SHRSP compared to 24-week WKY. In addition, significant difference according to age consistent with higher expression of GFAP in 24-week SHRSP compared to 7-week SHRSP was also found. GFAP: Glial fibrillary acidic protein; Iba1: ionized calcium binding adaptor molecule 1; SHRSP: spontaneously hypertensive stroke-prone rats; WKY: Wistar Kyoto rats; ns: not statistically significant; *: *p* < 0.05; **: *p* < 0.01; ***: *p* < 0.001; ****: *p* < 0.0001. Data represent the means and standard errors.

Since expression of CD38 was observed on microglia and glial cells, we examined the change in expression of Iba1 and GFAP in WKY and SHRSP as measures of microglial and glial cell activation, respectively (Figure 7). ANOVA testing of Iba1 expression showed significant interaction between rat age and type (*p* = 0.0035). In addition, the main effect of rat age and type were also significant (*p* = 0.0015, *p* < 0.0001). Group comparisons are presented in Figure 7B showing 98% higher expression in 24-week SHRSP vs. 24-week WKY (*p* < 0.0001) and 58% higher expression in 24-week SHRSP vs. 7-week SHRSP (*p* < 0.0001). There was a trend towards significance in the difference of Iba1 expression in 7-week SHRSP vs. 7-week WKY (31% higher in SHRSP, *p* = 0.07). In the case of GFAP expression (Figure 7C), there was no interaction between rat age and type using ANOVA (*p* = 0.49). But a significant main effect of age (*p* = 0.035) and rat type (*p* = 0.0004) were seen. Results of group comparisons are presented in Figure 7D. GFAP expression was 41% higher in 7-week SHRSP vs. 7-week WKY (*p* = 0.025) and 51% higher in 24-week SHRSP vs. 24-week WKY (*p* = 0.0026). GFAP expression also 25% higher in 24-week SHRSP vs. 7-week SHRSP (*p* = 0.045).

## Discussion

In this study, we demonstrate that CD38 expression and enzymatic activity are increased in the brains of SHRSP in comparison to control WKY. This can be seen in SHRSP at 7 weeks of age prior to the development of significant CSVD lesions and it persists at older age when CSVD lesions are clearly seen. In association, the brains of SHRSP exhibit lower levels of NAD(P)H and NO with higher superoxide generation compared to WKY. In addition, there were increases in CD38 expression with age in WKY and SHRSP and an increase in CD38 enzymatic activity in SHRSP with age as well. These results suggest that SHRSP, a genetic model of hypertension and human CSVD, exhibit increased expression and enzymatic activity of CD38 with functional consequences in the brain of these rats. To our knowledge, this is the first study to examine CD38 expression and enzymatic activity in SHRSP and control WKY.

CD38 is a glycoprotein that mainly exists in the cytoplasmic membrane of the cells, and it has a short cytoplasmic tail, transmembrane domain, and extracellular domain (Hogan et al., 2019; Guerreiro et al., 2020). CD38 biological function is complex, and it relates to its roles as an ectoenzyme and a receptor (Hogan et al., 2019; Guerreiro et al., 2020). As an ectoenzyme, the main function of CD38 is as an NADase that catabolizes β-NAD^+^ and its extracellular precursors nicotinamide mononucleotide (NMN) and nicotinamide riboside (NR) prior to their transport into the cells for NAD^+^ biosynthesis (Camacho-Pereira et al., 2016). This suggests that the primary role of CD38 is to maintain NAD^+^ homeostasis by regulating the synthesis of its precursors in the extracellular space. The other enzymatic role of CD38 involves the synthesis of cADPR (Guerreiro et al., 2020). The receptor role of CD38 is less characterized. However, it has been suggested that CD38 receptor function influences CD38 enzyme activity through its pH-dependency (Fang et al., 2018). Furthermore, CD31 may serve as CD38 ligand and mediate its internalization (Guerreiro et al., 2020). The latter is of potential interest in the brain since CD31 is primarily expressed on the endothelial cells that form the blood brain barrier (BBB) (Wimmer et al., 2019). Another role of CD38 receptor has been described in activated cells, mainly T lymphocytes, where it regulates cell adhesion and cooperates in signal transduction mediated by major receptor complexes (Morandi et al., 2019).

These complex receptor and enzymatic roles of CD38 have led researchers to implicating it in several diseases primarily related to inflammation, immunomodulation and cancer (Hogan et al., 2019). Importantly, CD38 enzymatic function has been implicated in age-related NAD^+^ decline (Camacho-Pereira et al., 2016). In this recent work, the loss of function of CD38 protected against age-related NAD^+^ decline and mitochondrial dysfunction, while the level and enzymatic activity of CD38 increased with aging (Camacho-Pereira et al., 2016). These recent findings have encouraged further research into the role of CD38 in various age-related neurological diseases (Guerreiro et al., 2020). Indeed, previous studies have found that the loss of CD38 enzymatic function was protective against Alzheimer’s Disease (AD) pathologies in a model of AD-prone CD38-deficient mice (Blacher et al., 2015). Furthermore, in another experiment in a mouse model of demyelination, deletion of CD38 suppressed glial activation and neuroinflammation (Roboon et al., 2019). Finally, the loss of CD38 function has similarly shown protection against ischemic brain injury in a previous study (Long et al., 2017). However, the role of CD38 in hypertension induced vascular cognitive impairment and cerebral small vessel disease has not yet been investigated.

Hypertension is the leading cardiovascular risk factor for intracerebral hemorrhage, vascular cognitive impairment, and acute ischemic stroke of lacunar type (Oparil et al., 2018). The brains of elderly patients with hypertension show vascular changes consistent with CSVD that accumulate with age including enlarged perivascular spaces, demyelination, microbleed formation and lacunes (Wardlaw et al., 2013). To identify changes of CD38 expression and enzymatic activity in the brain in the setting of hypertension, we utilized spontaneously hypertensive stroke-prone rats (SHRSP), an established genetic model for severe hypertension (Nabika et al., 2012). The brain of SHRSP shows histopathological evidence like human CSVD including microbleed formation, enlarged perivascular spaces and vascular wall thickening (Bailey et al., 2011a; Schreiber et al., 2012; Hannawi et al., 2021b). In the current study, we selected 7- and 24-week-old rats based on our prior research since most rats are phenotypically normal at 7 weeks of age prior to the development of hypertension, while they develop histological changes of the brain that are consistent CSVD lesions at 24 weeks of age (Hannawi et al., 2021b). In addition to the histopathological evidence of CSVD in the old SHRSP, the brains of SHRSP also exhibit evidence for endothelial dysfunction, BBB breakdown, neuroinflammation and oxidative stress even starting at younger age (Miyazaki et al., 2002; Bailey et al., 2011b). Our current results are consistent with this prior literature showing elevated superoxide level, lower NO with decreased eNOS and nNOS expression in the brains of SHRPS compared to age matched WKY. These findings provide evidence for ongoing oxidative stress in the brain of SHRSP with associated effect on NOS function manifested by the lower NO availability with decrease in eNOS and nNOS expression. In addition, the higher expression of iNOS, Iba1 and GFAP point toward neuroinflammation with evidence for microglial activation and astrogliosis.

The source of superoxide in the brain of SHRSP is likely multifactorial. Previous studies have suggested important roles for NADPH oxidase as its main source (Michihara et al., 2016). Additional sources include inflammation and NOS uncoupling as potentially seen in our data. We hypothesize that the oxidative stress leads to the observed increase in CD38 enzymatic activity which may potentially in turn result in the vicious cycle of worsening of oxidative stress with additional CD38 activation. We postulate this hypothesis based on our prior data in coronary ischemia/reperfusion models where we identified that oxidative stress associated with coronary reperfusion results in significant increase in CD38 enzymatic activity and NAD(P)H depletion leading to endothelial dysfunction (Reyes et al., 2015). Furthermore, the genetic deletion of CD38 conferred protection against ischemic heart injury (Boslett et al., 2018a). The protective role of CD38 loss of function against oxidative injury and endothelial dysfunction in coronary ischemia/reperfusion was further demonstrated by the pharmacologic administration of potent CD38 inhibitor in additional experiments (Boslett et al., 2019). We postulate that the oxidative stress and inflammation present in the brain of SHRSP leads to increased CD38 enzymatic activity with functional consequences of decrease in NAD(P)H levels. Indeed, we observed NAD(P)H depletion in the brain of these rats. This hypothesis, however, needs to be confirmed in future mechanistic studies that evaluate the role of oxidant stress and inflammation as triggers of CD38 activation in the brain.

Since the brain is comprised of several cell types, we performed studies to measure and map CD38 expression in these different cell types. We detected CD38 expression on endothelial cells, astrocytes, and microglia. There are a few prior studies that investigated the expression of CD38 in the brain of mice, rats, and humans (Mizuguchi et al., 1995; Yamada et al., 1997; Ceni et al., 2003; Braidy et al., 2014). In the one study of human brain that included 4 control subjects, CD38 was found to be expressed in the perikarya and dendrites of many neurons in the cortex and cerebellar Purkinje cells. This study, however, did not assess CD38 expression in the astrocytes or microglia (Mizuguchi et al., 1995). In the rat brain, two studies assessed CD38 expression in Wistar rats (Yamada et al., 1997; Braidy et al., 2014). The first study addressed the ultrastructural localization of CD38 using immunoelectron microscopy in the cerebral cortex and cerebellum. The findings of this study identified CD38 immunoreactivity in a subset of pyramidal neurons in the perikarya and dendrites in the cortex and several neuron types in the cerebellum. Immuno-reactivity was also found in the astrocytes as well. However, oligodendrocytes and microglia were reported to be immunonegative for CD38 (Yamada et al., 1997). In another study of aging Wistar rats, CD38 was found to be the main regulator of NAD^+^ levels in the neurons with aging. However, this study did not address CD38 expression in the different cellular subtypes of the brain (Braidy et al., 2014). It is noteworthy to mention that both studies assessed CD38 expression in normal aging control Wistar rats.

In comparison, in our study, we aimed to assess CD38 expression in the brain in presence of hypertension using SHRSP which are a known genetic model for marked hypertension and CSVD. Our findings point towards strong expression of CD38 on astrocytes, microglia, and endothelial cells. In association, SHRSP showed evidence for microglia activation and astrogliosis as evidenced by their increased expression of Iba-1 and GFAP, respectively. We postulate that microglia and astrocytes when activated overexpress CD38 which may lead to functional consequences on brain metabolism as evident by the decreased NAD(P)H levels and increased oxidative stress markers measured. Finally, CD38 expression on the endothelial cells in the brain of SHRSP may also suggest its potential role in endothelial cell dysfunction in the setting of hypertension as evident by the decreased NO and eNOS levels observed in our experiments. These findings are in line with our previous research showing that dysfunctional endothelial cells in the setting of cardiac ischemia also express CD38 that is activated with associated impairment of eNOS function (Boslett et al., 2018b).

Future research will be needed to address several additional remaining important questions. First, we observed that CD38 expression and enzymatic activity are increased in SHRSP at 7 weeks of age prior to onset of significant CSVD. However, studying earlier time points in the life of SHRSP is important to determine the onset and timeline of this elevation. Second, while the main goal of this work is to describe CD38 expression and enzymatic activity in the brain of SHRSP, additional future research is needed to mechanistically confirm this role through using specific CD38 inhibitors that aim to reverse the decrease in NAD(P)H and potentially decrease the associated oxidative stress process. This will be a subject of future investigation. Finally, we used male rats in this experiment as they have been shown to have higher blood pressure and consistent CSVD. Future studies will also need to evaluate the expression and enzymatic activity in female SHRSP to determine whether differences may exist from the males.

In conclusion, we show that SHRSP, a genetic model of marked hypertension and CSVD, exhibits increased CD38 expression and enzymatic activity in the brain compared to normotensive control WKY. Associated with this, the brain of SHRSP exhibits signs of oxidative stress, impaired NO availability, decreased eNOS expression, and neuroinflammation. Our findings suggest the potential role of CD38 in the pathogenesis of hypertension-induced CSVD and indicate the importance of future studies aiming to inhibit the enzymatic activity of CD38 to prove this role and develop future disease targeted therapeutics.

## Data Availability

The raw data supporting the conclusions of this article will be made available by the authors upon request, without undue reservation.
