# Supplementary figures and images for: Characterizing CD38 Expression and Enzymatic Activity in the Brain of Spontaneously Hypertensive Stroke-Prone Rats
Source: Front Pharmacol. 2022 May 31;13:881708. doi: 10.3389/fphar.2022.881708 (PMC9194821; doi:10.3389/fphar.2022.881708)

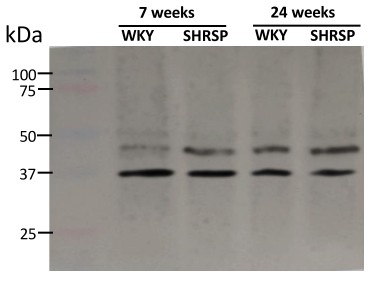

Supplement: Supplementary file 1 [file Image2.TIF]

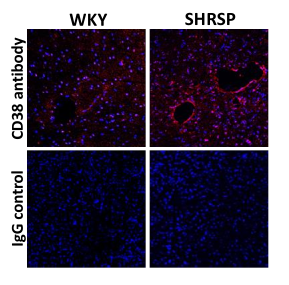

Supplement: Supplementary file 2 [file Image1.TIF]
