# Supplementary material for: Characterizing CD38 Expression and Enzymatic Activity in the Brain of Spontaneously Hypertensive Stroke-Prone Rats
Source: Front Pharmacol. 2022 May 31;13:881708. doi: 10.3389/fphar.2022.881708 (PMC9194821; doi:10.3389/fphar.2022.881708)
Supplement: Supplementary file 3 [file DataSheet1.PDF]

Supplementary Table 1: List of antibodies and chemicals in addition to their sources and concentrations that were used in our experiments:

Antibody/Chemical	Company Source	Concentration /Amount	Company Catalogue Number
CD68	Abcam	1:200	ab125212
CD3	Abcam	1:200	ab16669
GFAP	Abcam	1:200	ab7260
NeuN (neuronal marker)	Abcam	1:200	ab104225
Iba1	FUJIFILM Wako Chemicals	1:200	019-19741
CD38	Santa Cruz	1:25 in immunohistochemistry<br>1:100 Western Blotting	sc-374650
nNOS	Cell Signaling	1:200	4231S
MPO	Abcam	1:200	Ab208670
eNOS	Invitrogen	1:100	PA1-037
iNOS	Invitrogen	1:50	PA1-036
GAPDH	Cell Signaling Technology	1:2000	2118
Anti-mouse IgG, HRP-linked antibody	Cell Signaling Technology	1:5000	7076
Normal mouse IgG	Santa Cruz	1:25	Sc-2025
Goat anti-Mouse IgG (H+L) Cross-Adsorbed Secondary Antibody, Alexa Fluor 594	Invitrogen	1:500	A-11005
Goat anti-Rabbit IgG (H+L) Cross-Adsorbed Secondary Antibody, Alexa Fluor 488	Invitrogen	1:500	A-11008
DHE stain Dihydroethedium	Sigma-Aldrich	10 uM	104821-25-2
DAF-FM stain 4-Amino-5-methylamino-2',7'-difluorofluorescein diacetate, Diaminofluorescein-FM diacetate	Chemodex Ltd.	10 uM	254109-22-3
Cell Meter™ Intracellular NADH/NADPH Fluorescence Imaging Kit	AAT Bioquest, Inc.	1:250	15290

Supplementary Figure 1 legend:

Supplementary figure 1 shows the specificity of CD38 antibody that was used in the immunohistochemistry experiment in both WKY and SHRSP with positive red staining. Matched experiments with normal mouse IgG control show negative staining. Red: CD38 staining, Blue: DAPI: 4′,6-diamidino-2-phenylindole; SHRSP: spontaneously hypertensive stroke-prone rat; WKY: Wistar Kyoto Rat.

Supplementary Figure 2 legend:

Supplementary figure 2 shows the full western blot results demonstrating CD38 and GAPDH bands. GAPDH was detected at 37kD and CD38 band was detected at 43kD in WKY and SHRSP. SHRSP: spontaneously hypertensive stroke prone rat; WKY: Wistar Kyoto rat.
